# Predictive Value of Non-high-Density Lipoprotein Cholesterol and Neutrophil-Lymphocyte Ratio for Coronary Artery Vulnerable Plaques in Type 2 Diabetes Mellitus

**DOI:** 10.3389/fcvm.2022.927768

**Published:** 2022-06-20

**Authors:** Xiyi Huang, Shaomin Yang, Qiang Zhao, Xinjie Chen, Jialing Pan, Shaofen Lai, Fusheng Ouyang, Lingda Deng, Yongxing Du, Xiaohong Li, Qiugen Hu, Baoliang Guo, Jiemei Liu

**Affiliations:** ^1^Department of Clinical Laboratory, The Affiliated Shunde Hospital of Guangzhou Medical University, Foshan, China; ^2^Department of Radiology, The Affiliated Shunde Hospital of Guangzhou Medical University, Foshan, China; ^3^Department of Cardiovascular Medicine, The Affiliated Shunde Hospital of Guangzhou Medical University, Foshan, China; ^4^Department of Radiology, Shunde Hospital, Southern Medical University (The First People’s Hospital of Shunde, Foshan), Foshan, China; ^5^Department of Rehabilitation Medicine, Shunde Hospital, Southern Medical University (The First People’s Hospital of Shunde, Foshan), Foshan, China

**Keywords:** type 2 diabetes mellitus, coronary heart disease, coronary computed tomography angiography, vulnerable plaque, neutrophil-lymphocyte ratio, non-high-density lipoprotein cholesterol

## Abstract

**Background:**

Patients with diabetes have an increased risk of developing vulnerable plaques (VPs), in which dyslipidemia and chronic inflammation play important roles. Non-high-density lipoprotein cholesterol (non-HDL-C) and neutrophil-lymphocyte ratio (NLR) have emerged as potential markers of both coronary artery VPs and cardiovascular prognosis. This study aimed to investigate the predictive value of non-HDL-C and NLR for coronary artery VPs in patients with type 2 diabetes mellitus (T2DM).

**Methods:**

We retrospectively enrolled 204 patients with T2DM who underwent coronary computed tomography angiography between January 2018 and June 2020. Clinical data including age, sex, hypertension, smoking, total cholesterol, low-density lipoprotein cholesterol, HDL-C, triglyceride, non-HDL-C, glycated hemoglobin, neutrophil count, lymphocyte count, NLR, and platelet count were analyzed. Multivariate logistic regression was used to estimate the association between non-HDL-C, NLR, and coronary artery VPs. Receiver operating curve analysis was performed to evaluate the value of non-HDL-C, NLR, and their combination in predicting coronary artery VPs.

**Results:**

In our study, 67 patients (32.84%) were diagnosed with VPs, 75 (36.77%) with non-VP, and 62 (30.39%) with no plaque. Non-HDL-C and NLR were independent risk factors for coronary artery VPs in patients with T2DM. The areas under the ROC curve of non-HDL-C, NLR, and their combination were 0.748 [95% confidence interval (CI): 0.676–0.818], 0.729 (95% CI: 0.650–0.800), and 0.825 (95% CI: 0.757–0.887), respectively.

**Conclusion:**

Either non-HDL-C or NLR could be used as a predictor of coronary artery VPs in patients with T2DM, but the predictive efficiency and sensitivity of their combination would be better.

## Introduction

Type 2 diabetes mellitus (T2DM) is one of the most prevalent chronic non-communicable diseases worldwide, with an estimated 415 million people aged 20–79 years having diabetes worldwide, according to 2017 data from the International Diabetes Federation (1, 2). Diabetic vascular diseases are the most common and serious chronic complications of diabetes, and coronary heart disease (CHD) is one of the leading causes of death in patients with T2DM. Recently, large scale meta-analyses showed that in individuals with mild elevated hyperglycemia, the risk of cardiovascular disease and worse prognosis was increased (3–6). The pathological basis of CHD is the presence of vulnerable plaques (VPs). When ruptured, they can cause secondary thrombosis, resulting in acute severe stenosis or occlusion of the lumen and ultimately resulting in clinical acute coronary events (7). Therefore, early identification of VPs is crucial to preventing cardiovascular events.

Atherosclerosis is a long-term chronic inflammatory process, novel inflammatory biomarkers may be useful for evaluation of the severity and prognosis of CHD (8, 9). Recently, the neutrophil-lymphocyte ratio (NLR) has been considered as a marker for chronic inflammation (10). NLR reflects the balance between neutrophils and lymphocytes in the body and can, therefore, relate to the systemic inflammatory response. Given its relationship with inflammation, NLR is considered a novel marker for the clinical prediction of cardiovascular events (11).

Patients with T2DM often present with mixed dyslipidemia, and low treatment compliance rate, which are risk factors for complicated CHD. Although lipid-lowering therapy can control the plasma low-density lipoprotein cholesterol (LDL-C) level in patients with CHD and reduce the risk of cardiovascular events, recent studies have shown that traditional lipid indexes such as LDL-C cannot fully reflect the actual scenario of lipid metabolism in CHD patients with T2DM. Therefore, novel and more specific lipid metrics related to cardiovascular disease pathology are needed to predict cardiovascular events better, and non-HDL-C is considered to play an essential role in the formation and development of coronary atherosclerosis.

Coronary artery computed tomography angiography (CCTA) can rapidly and accurately assess the degree of coronary artery stenosis and identify the morphology and components of coronary atherosclerotic plaques. VPs diagnosed by CCTA are highly consistent with pathology and have good predictive value for future cardiovascular events (12, 13), making their detection feasible in studies on the VPs of coronary atherosclerosis. Although a few previous studies have shown a close relationship between NLR or non-HDL-C and coronary artery VPs, atherosclerosis due to diabetes is different from atherosclerosis caused by other risk factors (14). Research on NLR or non-HDL-C and CCTA imaging in relation to T2DM is scarce.

Therefore, this study was conducted to explore whether the correlation between NLR, non-HDL-C, and VPs of coronary artery in patients with T2DM using CCTA. The possibility of using coronary artery VPs as a predictive marker of cardiovascular events in patients with T2DM was also explored.

## Materials and Methods

### Patient Selection and Grouping

We retrospectively collected data from 213 patients diagnosed with T2DM and examined by CCTA in Shunde Hospital of Southern Medical University between January 2018 and June 2020. The inclusion criteria were as follows: (1) Patients who met the 2017 American Diabetes Association diagnostic criteria (15); (2) Patients who were diagnosed with T2DM more than 5 years ago; (3) Patients who received CCTA and completed the VPs assessment; (4) Patients with complete clinical data. The exclusion criteria were as follows: (1) Patients who were diagnosed with type 1 diabetes or other types of diabetes or acute complications of diabetes; (2) Patients with acute infection; (3) Patients with severe cardiac insufficiency, arrhythmia, and acute myocardial infarction; (4) Patients with severe valvular heart disease, cardiomyopathy, rheumatic heart disease, congenital heart disease, severe liver insufficiency (Child-Pugh class C, or Alanine aminotransferase, ALT>250 U/L, or Total bilirubin, TBil>115 μ mol/L), severe renal insufficiency (glomerular filtration rate, RGF<30 mL/min), and malignant tumors; (5) Patients with poor quality of CCTA image that was insufficient for further analysis. According to plaque and plaque vulnerability, patients were divided into the no plaque group, non-vulnerable plaque group, and VP group. All patients provided written informed consent, and the local ethics committee approved the study.

### Scanning Protocol

All patients were scanned by a dual-source CT (SOMATOM Definition Flash, Siemens, Germany). Contrast-enhanced CT imaging was performed after 40 s delay following intravenous administration of 70 mL of iodinated contrast material (Ultravist 350, Bayer Schering Pharma, Berlin, Germany) at a rate of 5.0 mL/s with a pump injector (Ulrich CT Plus 150, Ulrich Medical, Ulm, Germany) after routine pre-contrast CT, followed by infusion of 50 mL of saline at the same infusion rate. The parameters were as follows: 120 kV, 90 kV; 320 mAs; rotation time, 0.33 s; detector collimation: 32 mm × 2 mm × 0.6 mm; pitch = 0.20–0.28 mm (automatic adjustment according to heart rate changes); slice thickness = 0.75 mm; slice gap = 0.5 mm; field of view (FOV) = 260 × 260 mm; matrix = 512 × 512.

### Imaging Processing and Analysis

All CCTA scans were evaluated for the presence of non-evaluable segments. According to the American Heart Association classification, coronary arteries were divided into 16 segments (16). Coronary plaques were defined as structures of at least 2 mm^2^ areas within and/or adjacent to the artery lumen, clearly distinguishable from the vessel lumen, and surrounded by pericardial tissue.

A Siemens post-processing workstation (Syngo.Via VB10, Siemens, Germany) was used to reconstruct coronary arteries for all patients. Maximum intensity projection, curved planar reformation (CRP), volume rendering, and other post-processing methods were used to analyze the images by radiologists. The imaging features of VPs include spotty calcification, positive reconstruction, low attenuation plaque, and napkin ring sign (NRS) (17). CCTA imaging data were analyzed and evaluated independently by two radiologists with more than 10 years of experience in cardiovascular disease imaging. Disagreements between the radiologists were resolved by consensus and, if necessary, by consultation with a third radiologist.

### Clinical Characteristics

The following clinical characteristics were determined: age, sex, height, weight, hyperlipidemia history, hypertension history (blood pressure>140/90 mmHg), smoking history, and drinking history. Blood samples were obtained from fasting venous blood for 8–12 h on the next morning. An automated blood cell counter (XE-2100, Sysmex, Kobe, Japan) was used for analysis according to the instructions, and NLR was calculated from the results. Biochemical indexes including total cholesterol (TC), TG, LDL-C, high-density lipoprotein cholesterol (HDL-C), and HbA1c (%) were measured using an automatic biochemical analyzer (Cobas-8000, Roche, Basel, Switzerland). Non-HDL-C was calculated by the formula (18): Non-HDL-C = TC–LDL-C. Two independent radiologists retrospectively reviewed the clinical characteristics with more than 10 years of experience in cardiovascular disease imaging.

### Statistical Analysis

SPSS26.0 and R software 3.60^1^ were used for statistical analysis. The Shapiro-Wilk test was used to ascertain the normality of the measurement data, which are expressed as mean ± standard deviation (X ± S). One-way ANOVA was used for statistical analysis of variance, and the Bonferroni correction method was used to compare the three groups. Statistical data are expressed as percentages, and statistical analysis was conducted using the χ^2^-test/Fisher’s exact test. Independent risk factors for VPs were obtained by univariate and multivariate logistic regression. The receiver operating characteristic (ROC) curve and the area under the ROC curve (AUC) were used to evaluate the predictive value. A *P*-value < 0.05 was considered to indicate statistical significance.

## Results

### Patients and Clinical Characteristics

After excluding nine patients (four due to missing image data, three due to allergy to contrast agent, and two due to phobia at the time of examination), 204 patients diagnosed with T2DMwere selected (mean age 63.1 ± 9.8 years; range, 34–85 years), and 103 (50.49%) were males. VPs was seen in 67 patients (32.84%), non-VP in 75 (36.77%), and no plaque in 62 patients (30.39%). Univariate analysis showed significant differences in age, hypertension, TC, LDL-C, HDL-C, TG, non-HDL-C, neutrophil count, and NLR among the three groups. Comparisons of patient clinical characteristics between the three groups are shown in Table 1.

**TABLE 1:** Baseline clinical characteristics for T2DM patients.

The patients’ baseline characteristics				
Characteristics	Vulnerable plaque	Non-vulnerable plaque	No plaque	*P*
	**(*n* = 67)**	**(*n* = 75)**	**(*n* = 62)**	
Age, mean ± *SD*, years	65.3 ± 8.8^a^	66.0 ± 9.2^a^	60.3 ± 10.5	0.001
**Sex, no. (%)**				
Male	37 (35.92)	37 (35.92)	29 (28.16)	0.612
Female	30 (29.71)	38 (37.62)	33 (32.67)	
Hypertension, no. (%)	52 (34.90)	60 (40.27)^a^	37 (24.83)	0.017
Smoking, no. (%)	13 (52.00)	6 (24.00)	6 (24.00)	0.089
Total cholesterol (mg/dl)	208.7 ± 38.7^ab^	189.4 ± 46.4	174.0 ± 46.4	< 0.001
LDL-C (mg/dl)	123.7 ± 34.8^ab^	104.4 ± 34.0	92.7 ± 31.3	< 0.001
HDL-C (mg/dl)	47.9 ± 10.4^a^	52.2 ± 31.3	57.2 ± 13.1	< 0.001
Triglycerides (mg/dl)	184.3 ± 108.9^b^	134.6 ± 78.9	147.0 ± 92.1	0.006
Non-HDL-C (mg/dl)	160.8 ± 39.5^ab^	137.2 ± 39.6	116.8 ± 34.6	< 0.001
HbA1c (%), mean ± *SD*	6.7 ± 1.4	6.9 ± 0.8	6.5 ± 0.8	0.063
Neutrophil (10^3^/μl)	4.89 ± 1.83^ab^	4.21 ± 1.24	4.00 ± 1.63	0.003
Lymphocyte (10^3^/μl)	1.92 ± 0.53	1.93 ± 0.65	1.99 ± 0.59	0.764
NLR (%), mean±*SD*	3.06 ± 1.36	2.24 ± 1.15	2.01 ± 0.79	< 0.001
Platelet (10^3^/μl)	205.20 ± 56.31^ab^	210.23 ± 67.60	211.29 ± 69.04	0.846
FBG (mmol/L)	7.44 ± 2.29	6.88 ± 2.41	6.46 ± 2.02	0.108
Insulin (*n*, %)	17 (25.4%)	17 (22.7%)	20 (32.3%)	0.213
OADs (*n*, %)	26 (38.8%)	32 (42.7%)	36 (58.1%)	0.068
Statins (*n*, %)	39 (58.2%)^b^	42 (56.0%)^b^	44 (71.0%)	0.165

*^a^P means compared with non-plaque group.*

*^b^P means compared with the non-vulnerable plaque group. NLR, neutrophil-lymphocyte ratio; SD, standard deviation; FBG, fasting blood glucose.*

### Risk Factors for Vulnerable Plaques

**FIGURE 1:**
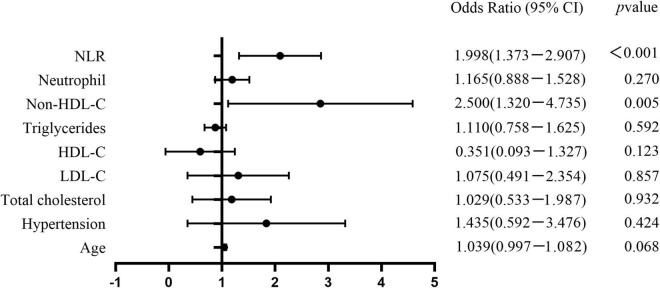
Forest plots show the comparison of risk factors for coronary artery vulnerable plaques in T2DM patients. Non-HDL-C (OR 2.500, 95%CI: 1.32–4.735) and NLR (OR 1.998, 95%CI: 1.373–2.907) were independent risk factors for coronary artery vulnerable plaques.

Multivariate logistic regression analysis was performed considering indexes found significant in the univariate analysis as independent variables. These included age, hypertension, TC, LDL-C, HDL-C, TG, non-HDL-C, neutrophil count, and NLR. Non-HDL-C [odds ratio (OR): 2.500, 95% CI: 1.32–4.735] and NLR (OR: 1.998, 95% CI: 1.373–2.907) were independent risk factors for VPs (Table 2 and Figure 1).

**TABLE 2:** Multifactorial logistic regression analysis of coronary artery vulnerable plaques in T2DM patients.

Variables	*B*	*S*.*E*	*WaldX* ^2^	*P*	*OR*	95% CI
**Multivariable analysis**						
Age	0.038	0.021	3.32	0.068	1.039	0.997–1.082
Hypertension	0.361	0.451	0.64	0.424	1.435	0.592–3.476
Total cholesterol	0.029	0.336	0.007	0.932	1.029	0.533–1.987
LDL-C	0.072	0.4	0.032	0.857	1.075	0.491–2.354
HDL-C	–1.046	0.678	2.382	0.123	0.351	0.093–1.327
Triglycerides	0.104	0.194	0.288	0.592	1.110	0.758–1.625
Non-HDL-C	0.916	0.326	7.908	0.005	2.500	1.32–4.735
Neutrophil	0.153	0.138	1.217	0.27	1.165	0.888–1.528
NLR	0.692	0.191	13.077	< 0.001	1.998	1.373–2.907

*NLR, neutrophil-lymphocyte ratio; OR, odds ratio; CI, confidence interval.*

### Diagnostic Performance of Different Lipid Indexes for Vulnerable Plaques

**FIGURE 2:**
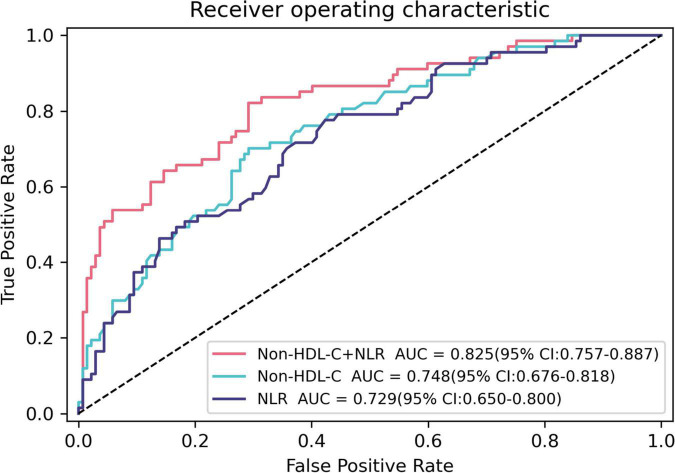
Receiver operating characteristic curves for predicting coronary artery vulnerable plaques using baseline Non-HDL-C, NLR, and their combination in T2DM patients. The AUC of Non-HDL-C, NLR, and their combination were 0.748 (95% CI: 0.676–0.818), 0.729 (95% CI: 0.650–0.800), and 0.825 (95% CI: 0.757–0.887), respectively.

Table 3 and Figure 2 show the ROC curves of non-HDL-C, NLR, and their combination in predicting coronary artery VPs in patients with T2DM. Non-HDL-C combined with NLR achieved the highest performance, with AUC 0.825 (95% CI: 0.757–0.887), sensitivity 82.1%, and specificity 70.8%, followed by non-HDL-C, with AUC 0.748 (95% CI: 0.676–0.818), sensitivity 0.701, and specificity 0.708, and NLR, with AUC 0.729 (95% CI: 0.650–0.800, sensitivity 0.776, and specificity 0.577).

**TABLE 3:** Predictive value of different lipid indicators for vulnerable plaques.

	Accuracy	Sensitivity	Specificity	*P*	F1_score	AUC (95% CI)
Non-HDL-C+NLR	0.745	0.821	0.708	0.003^a^	0.679	0.825 (0.757–0.887)
Non-HDL-C	0.706	0.701	0.708	0.697^b^	0.61	0.748 (0.676–0.818)
NLR	0.642	0.776	0.577	0.001^c^	0.588	0.729 (0.650–0.800)

*NLR, neutrophil-lymphocyte ratio; AUC, area under the curve; CI, confidence interval.*

*^a^P means compared with Non-HDL-C.*

*^b^P means compared with NLR.*

*^c^P means compared with Non-HDL-C+NLR.*

## Discussion

We identified two risk factors associated with coronary artery VPs in patients with T2DM: non-HDL-C concentration and NLR. HDL-C is an independent protective factor. Non-HDL-C combined with NLR achieved the best predictive performance, with an AUC 0.825 (95% CI: 0.757–0.887). Non-HDL-C and NLR have been shown to be of clinical value in predicting coronary artery VPs in patients with T2DM.

Epidemiological statistics have shown that CHD is the main cause of death among cardiovascular diseases. However, its pathogenesis is not completely understood, and the most common causes are endothelial cell injury, inflammatory reaction, hemodynamic changes, lipid metabolism disorder, immune factors, and genetic factors. DM is a risk factor for CHD. The incidence and mortality of cardiovascular events in patients with diabetes are much higher than those in the general population. Hyperglycemia can directly damage the intima layer of blood vessels, resulting in the deposition of LDL-C and other lipid substances in the intima and the activation of various inflammatory cells. Subsequently, neutrophils secrete inflammatory mediators such as interleukin-6 (IL-6) and tumor necrosis factor-α (TNF-α), which attract smooth muscle cells and macrophages for phagocytosis, resulting in vascular endothelial dysfunction (19, 20), and anti-inflammatory medicine or cytokines can improved the vascular endothelial dysfunction and decreased the risk of CHD (21, 22). Atherosclerotic plaques gradually form on the vascular wall. In the United States, about 25% of patients with CHD over 35 years old develop complications of diabetes (23). Patients with diabetes carry a 2–4 times greater risk of CHD than patients without diabetes, and about 75% of deaths among patients with diabetes are caused by coronary artery ischemia (24, 25). Esposito et al. observed predominant VPs in patients with diabetes (26). They concluded that T2DM influences the type and stability of atherosclerotic plaques, and patients with T2DM might have atherosclerotic patterns different from those seen in patients without diabetes. Patients with diabetes mainly have elevated TG, low HDL-C, and insulin resistance (IR), which promotes inflammatory vascular injury and induces the occurrence and development of CHD (27). On the one hand, the decrease in HDL-C levels will undoubtedly lead to an increase in non-HDL-C levels, reverse the reduction of the TC effect, and increase the risk of VPs in patients with diabetes. On the other hand, the increase in TG leads to an increase in TRL, which is closely related to coronary artery calcification (28). Therefore, non-HDL-C should be added to routine lipid assessment to evaluate coronary atherosclerosis (28).

In our study, univariate analysis showed that age, hypertension, TC, LDL-C, HDL-C, TG, non-HDL-C, neutrophil, and NLR showed statistically significant differences, but only non-HDL-C and NLR showed statistically significant differences among the three groups in multivariate analysis (*P* < 0.05). Non-HDL-C (OR: 2.500, 95%:1.32–4.735) and NLR (OR: 1.998, 95%:1.373–2.907) are significant risk factors for coronary artery VPs in patients with T2DM. Lowering serum LDL-C levels can delay the progression of atherosclerotic plaque formation and induce plaque regression (29). However, some patients with high residual cardiovascular risk, such as diabetes, metabolic syndrome, and obesity, carry a significantly increased risk of plaque formation (30). By focusing only on LDL-C levels, those at high risk for T2DM may be overlooked, increasing the risk of CHD in these patients. Currently, European guidelines for dyslipidemia management recommend that the main target of lipid regulation in patients with T2DM is LDL-C<2.6 mmol/L, and the secondary target is non-HDL-C<3.4 mmol/L (31). Non-HDL-C is a better risk estimation indicator than LDL-C, particularly in patients with T2DM, higher levels of TG, and metabolic syndrome (32). Wu et al. found that non-HDL-C was a risk factor for VPs (33). Their results indicated that VPs should be assessed more carefully in patients with high levels of non-HDL-C so that atherosclerotic cardiovascular disease events can be prevented, aligning well with our research.

NLR was found to be another risk factor for coronary artery VPs in patients with T2DM. However, not many studies on NLR and coronary artery VPs, especially in patients with T2DM, have been conducted. A chronic inflammatory response is closely related to diabetic macrovascular lesions (34), and inflammatory response plays an essential role in the formation and rupture of VPs. In existing plaques, neutrophils gather around new or damaged plaques, promoting the release of inflammatory cytokines and activating monocytes to transform them into macrophages. This, in turn, accelerates the formation and shedding of new plaques. VPs in the coronary artery can rupture and lead to thrombosis, which can cause acute coronary syndrome (35). Under inflammatory conditions in patients with T2DM, the number of CD8+T lymphocytes is reduced, accompanied by the imbalance in lymphocyte function and subpopulation ratio, thereby decreasing immunity. Thus, chronic inflammation persists. Chronic low-grade inflammation eventually leads to IR and insulin secretion dysfunction, promoting the occurrence of T2DM and its complications (36). NLR is relatively constant compared to absolute counts of neutrophils and lymphocytes, reflecting a balance between inflammatory activators and inflammatory regulators (37). Yun et al. found that CAD patients with a high NLR are at a higher risk of developing VPs and extensive inflammation, leading to acute coronary events (38). They suggested that NLR can be used as a valuable tool to detect significant atherosclerosis and VPs in patients with CAD. Therefore, the early monitoring of NLR can directly affect the occurrence and development of coronary artery events in patients with T2DM, especially the occurrence and development of coronary artery VPs.

The ROC curve results of this study showed that the AUCs of non-HDL-C and NLR for predicting coronary VPs in patients with T2DM were 0.748 and 0.729, respectively, and the AUC of the combination of the two parameters for predicting coronary artery VPs in patients with T2DM was 0.825. The predictive value of non-HDL-C combined with NLR was significantly higher than that achieved when using non-HDL-C or NLR. Therefore, the detection of non-HDL-C and NLR in CAD patients with T2DM facilitates the evaluation of the vulnerability of plaques, thereby improving the prognosis and quality of life of patients.

This study was subject to several limitations. First, this was a retrospective study performed at a single center. Second, this study does not investigate the relationship between different VP types and non-HDL-C and NLR in patients with T2DM and CHD. Finally, a prospective study should be performed to validate the findings.

## Conclusion

In conclusion, this study demonstrates that elevated serum non-HDL-C and NLR were independent risk factors for coronary artery VPs in patients with T2DM, and elevated HDL-C is an independent protective factor. Both NLR and non-HDL-C can be used to predict the development of VPs in patients with T2DM, and their combination achieves better predictive efficacy, with higher sensitivity and accuracy, providing a reference basis for the early diagnosis and treatment of VPs of the diabetic coronary artery.

## Data Availability Statement

The raw data supporting the conclusions of this article will be made available by the authors, without undue reservation.

## Ethics Statement

The studies involving human participants were reviewed and approved by Medical Ethics Committee of Shunde Hospital, Southern Medical University. The patients/participants provided their written informed consent to participate in this study. Written informed consent was obtained from the individual(s) for the publication of any potentially identifiable images or data included in this article.

## Author Contributions

XH, SY, JL, BG, FO, and QH: conception and design. XH, QZ, XC, JP, SL, and FO: acquisition of data. XH, SY, BG, FO, LD, YD, XL, and QH: analysis and interpretation of data. XH, SY, BG, and JL: drafting or revising the article. All authors contributed to the article and approved the submitted version.

## Conflict of Interest

The authors declare that the research was conducted in the absence of any commercial or financial relationships that could be construed as a potential conflict of interest.

## Publisher’s Note

All claims expressed in this article are solely those of the authors and do not necessarily represent those of their affiliated organizations, or those of the publisher, the editors and the reviewers. Any product that may be evaluated in this article, or claim that may be made by its manufacturer, is not guaranteed or endorsed by the publisher.

## Footnotes

1
https://www.r-project.org/

## References

[1] Henning RJ. Type-2 diabetes mellitus and cardiovascular disease. *Future Cardiol.* (2018) 14:491–509. 30409037 10.2217/fca-2018-0045

[2] Ogurtsova K da Rocha Fernandes JD Huang Y Linnenkamp U Guariguata L Cho NH IDF diabetes Atlas:global estimates for the prevalence of diabetes for 2015 and 2040. *Diabetes Res Clin Pract.* (2017) 128:40–50. 10.1016/j.diabres.2017.03.024 28437734

[3] Cai X Zhang Y Li M Wu JH Mai L Li J Association between prediabetes and risk of all cause mortality and cardiovascular disease: updated meta-analysis. *BMJ.* (2020) 370:m2297. 10.1136/bmj.m2297 32669282 PMC7362233

[4] Huang Y Cai X Mai W Li M Hu Y. Association between prediabetes and risk of cardiovascular disease and all cause mortality: systematic review and meta-analysis. *BMJ.* (2016) 355:i5953. 10.1136/bmj.i5953 27881363 PMC5121106

[5] Cai X Liu X Sun L He Y Zheng S Zhang Y Prediabetes and the risk of heart failure: a meta-analysis. *Diabetes Obes Metab.* (2021) 23:1746–53. 10.1111/dom.14388 33769672

[6] Mai L Wen W Qiu M Liu X Sun L Zheng H Association between prediabetes and adverse outcomes in heart failure. *Diabetes Obes Metab.* (2021) 23:2476–83. 10.1111/dom.14490 34227220

[7] Stefanadis C Antoniou CK Tsiachris D Pietri P. Coronary atherosclerotic vulnerable plaque: current perspectives. *J Am Heart Assoc.* (2017) 6:e005543. 10.1161/JAHA.117.005543 28314799 PMC5524044

[8] Golia E Limongelli G Natale F Fimiani F Maddaloni V Pariggiano I Inflammation and cardiovascular disease: from pathogenesis to therapeutic target. *Curr Atheroscler Rep.* (2014) 16:435. 10.1007/s11883-014-0435-z 25037581

[9] Huang A Huang Y. Role of SFRPS in cardiovascular disease. *Ther Adv Chronic Dis.* (2020) 11:2040622320901990. 10.1177/2040622320901990 32064070 PMC6987486

[10] Bressi E Mangiacapra F Ricottini E Cavallari I Colaiori I Di Gioia G Impact of neutrophil-to-lymphocyte ratio and platelet-to-lymphocyte ratio on 5-year clinical outcomes of patients with stable coronary artery disease undergoing elective percutaneous coronary intervention. *J Cardiovasc Trans Res.* (2018) 11:517–23. 10.1007/s12265-018-9829-6 30276618

[11] Ahsen A Ulu MS Yuksel S Demir K Uysal M Erdogan M As a new inflammatory marker for familial mediterranean fever: neutrophil-to-lymphocyte ratio. *Inflammation.* (2013) 36:1357–62. 10.1007/s10753-013-9675-2 23794006

[12] Mandal SR Bharati A Haghighi RR Arava S Ray R Jagia P Non-invasive characterization of coronary artery atherosclerotic plaque using dual energy CT: explanation in ex-vivo samples. *Phys Med.* (2018) 45:52–8. 10.1016/j.ejmp.2017.12.006 29472090

[13] Conte E Annoni A Pontone G Mushtaq S Guglielmo M Baggiano A Evaluation of coronary plaque characteristics with coronary computed tomography angiography in patients with non-obstructive coronary artery disease: a long-term follow-up study. *Eur Heart J Cardiovasc Imaging.* (2017) 18:1170–8. 10.1093/ehjci/jew200 27679600

[14] Beckman JA Paneni F Cosentino F Creager MA. Diabetes and vascular disease: pathophysiology, clinical consequences, and medical therapy: part II. *Eur Heart J.* (2013) 34:2444–52. 23625211 10.1093/eurheartj/eht142

[15] Marathe PH Gao HX Close KL. American diabetes association standards of medical care in diabetes 2017. *J Diabetes.* (2017) 9:320–4. 28070960 10.1111/1753-0407.12524

[16] Austen WG Edwards JE Frye RL Gensini GG Gott VL Griffith LS A reporting system on patients evaluated for coronary artery disease. Report of the Ad HOC committee for grading of coronary artery disease, council on cardiovascular surgery, American heart association. *Circulation.* (1975) 51:5–40. 10.1161/01.cir.51.4.5 1116248

[17] Beg F Rehman H Al-Mallah MH. The vulnerable plaque: recent advances in computed tomography imaging to identify the vulnerable patient. *Curr Atheroscler Rep.* (2020) 22:58. 10.1007/s11883-020-00879-z 32772222

[18] Expert Panel on Detection, Evaluation, Treatment of High Blood Cholesterol in Adults. Executive summary of the third report of the national cholesterol education program (NCEP) expert panel on detection, evaluation, and treatment of high blood cholesterol in adults (adult treatment Panel III). *JAMA.* (2001) 285:2486–97. 10.1001/jama.285.19.2486 11368702

[19] Maiolino G Bisogni V Rossitto G Rossi GP. Lipoprotein-associated phospholipase A2 prognostic role in atherosclerotic complications. *World J Cardiol.* (2015) 7:609–20. 10.4330/wjc.v7.i10.609 26516415 PMC4620072

[20] Li D Zhao L Yu J Zhang W Du R Liu X Lipoprotein-associated phospholipase A2 in coronary heart disease: review and meta-analysis. *Clin Chim Acta.* (2017) 465:22–9. 27956130 10.1016/j.cca.2016.12.006

[21] Wu J Zheng H Liu X Chen P Zhang Y Luo J Prognostic value of secreted frizzled-related protein 5 in heart failure patients with and without type 2 diabetes mellitus. *Circ Heart Fail.* (2020) 13:e007054. 10.1161/CIRCHEARTFAILURE.120.007054 32842761

[22] Zheng S Zheng H Huang A Mai L Huang X Hu Y Piwi-interacting RNAs play a role in vitamin C-mediated effects on endothelial aging. *Int J Med Sci.* (2020) 17:946–52. 10.7150/ijms.42586 32308548 PMC7163353

[23] Juárez-Rojas JG Posadas-Romero C Martínez-Alvarado R Jorge-Galarza E Reyes-Barrera J Sánchez-Lozada LG Type 2 diabetes mellitus is associated with carotid artery plaques in patients with premature coronary heart disease. *Rev Invest Clin.* (2018) 70:301–9. 10.24875/RIC.18002591 30532096

[24] Sypalo A Kravchun P Kadykova O. The influence of mono— and multivascular lesions of coronary arteries on the course of coronary heart disease in patients with diabetes mellitus type 2. *Georgian Med News.* (2017) 264:61–5. 28480852

[25] Lyu Y Luo Y Li C Guo X Lu J Wu H Regional differences in the prevalence of coronary heart disease and stroke in patients with type 2 diabetes in China. *J Clin Endocrinol Metab.* (2018) 103:3319–30. 10.1210/jc.2018-00422 29982638

[26] Esposito L Saam T Heider P Bockelbrink A Pelisek J Sepp D MRI plaque imaging reveals high-risk carotid plaques especially in diabetic patients irrespective of the degree of stenosis. *BMC Med Imaging.* (2010) 10:27. 10.1186/1471-2342-10-27 21118504 PMC3004802

[27] Newman JD Schwartzbard AZ Weintraub HS Goldberg IJ Berger JS. Primary prevention of cardiovascular disease in diabetes mellitus. *J Am Coll Cardiol.* (2017) 70:883–93. 28797359 10.1016/j.jacc.2017.07.001 PMC5656394

[28] Bittencourt MS Santos RD Staniak H Sharovsky R Kondapally R Vallejo-Vaz AJ Relation of fasting triglyceride-richlipoprotein cholesterol to coronary artery calcium score (from the ELSA-Brasil study). *Am J Cardiol.* (2017) 119:1352–8. 10.1016/j.amjcard.2017.01.033 28302272

[29] Elshazly MB Stegman B Puri R. Regression of coronary atheroma with statin therapy. *Curr Opin Endocrinol Diabetes Obes.* (2016) 23:131–7. 26910274 10.1097/MED.0000000000000234

[30] Boden WE Bhatt DL Toth PP Ray KK Chapman MJ Lüscher TF. Profound reductions in first and total cardiovascular events with icosapent ethyl in the REDUCE-IT trial: why these results usher in a new era in dyslipidaemia therapeutics. *Eur Heart J.* (2020) 41:2304–12. 10.1093/eurheartj/ehz778 31872245 PMC7308541

[31] Mach F Baigent C Catapano AL Koskinas KC Casula M Badimon L 2019 ESC/EAS guidelines for the management of dyslipidaemias:lipidmodification to reduce cardiovascular risk. *Eur Heart J.* (2020) 41:111–88. 31504418 10.1093/eurheartj/ehz455

[32] Robinson JG Wang S Smith BJ Jacobson TA. Meta-analysis of the relationship between non-high-density lipoprotein cholesterol reduction and coronary heart disease risk. *J Am Coll Cardiol.* (2009) 53:316–22. 10.1016/j.jacc.2008.10.024 19161879

[33] Wu J Zhang J Wang A Chen S Wu S Zhao X. Association between non-high-density lipoprotein cholesterol levels and asymptomatic vulnerable carotid atherosclerotic plaques. *Eur J Neurol.* (2019) 26:1433–8. 10.1111/ene.13973 31002203

[34] Tuttolomondo A Maida C Pinto A. Diabetic foot syndrome:immune-inflammatory features as possible cardiovascular markers in diabetes. *World J Orthop.* (2015) 6:62–76. 10.5312/wjo.v6.i1.62 25621212 PMC4303791

[35] Virmani R Burke AP Farb A Kolodgie FD. Pathology of the vulnerable plaque. *J Am Coll Cardiol.* (2006) 47:C13–8. 16631505 10.1016/j.jacc.2005.10.065

[36] Luo A Leach ST Barres R Hesson LB Grimm MC Simar D. The microbiota and epigenetic regulation of T helper 17/regulatory T cells: in search of a balanced immune system. *Front Immunol.* (2017) 8:417. 10.3389/fimmu.2017.00417 28443096 PMC5385369

[37] Gibson PH Croal BL Cuthbertson BH Small GR Ifezulike AI Gibson G Preoperativeneutrophil-lymphocyte ratio and outcome from coronary artery bypassgrafting. *Am Heart J.* (2007) 154:995–1002. 10.1097/HJR.0b013e3280403c68 17967611

[38] Yun HC Young JH Youngkeun A Park IH Jeong MH. Relationship between neutrophil-to-lymphocyte ratio and plaque components in patients with coronary artery disease: virtual histology intravascular ultrasound analysis. *J Korean Med Sci.* (2014) 29:950–6. 10.3346/jkms.2014.29.7.950 25045227 PMC4101783

